# Hepatitis B virus infection among illegal drug users in Enugu State, Nigeria: prevalence, immune status, and related risk factors

**DOI:** 10.1186/s12889-024-18675-8

**Published:** 2024-05-09

**Authors:** Ibuchukwu N Orabueze, Anthony C Ike, Oluchi M Aniche, Ifeyinwa N Nwafia, Samuel O Ebede

**Affiliations:** 1 Department of Microbiology, Faculty of Biological Sciences, University of Nigeria, Nsukka, Enugu State Nigeria; 2 Department of Medical Microbiology, Faculty of Basic Clinical Sciences, College of Medicine, University of Nigeria Ituku-Ozalla Campus, Enugu, Enugu State Nigeria; 3 Department of Community Medicine, Faculty of Basic Clinical Sciences, University of Nigeria Enugu Campus, Enugu, Enugu State Nigeria

**Keywords:** Drug users, HBV, Illegal, Prevalence, Immunity, Risk factors, Nigeria

## Abstract

**Introduction:**

Hepatitis B virus infection poses a global health challenge, particularly in low- and middle-income African countries. Illicit drug use exacerbates the problem, with drug users having a higher HBV infection risk and maintaining a quiet transmission pool. This study aimed to determine HBV infection prevalence, immune status, and risk factors among illegal drug users in Enugu State, Nigeria.

**Materials and methods:**

A cross-sectional study was conducted in Enugu State, using privileged access interviewer methods to enroll drug users. Pre-tested structured questionnaires were administered after informed consent was obtained. Blood samples were tested for HBsAg, HBsAb, HBeAg, HBeAb, and HBcAb using immunochromatographic rapid test kits. Statistical analysis was performed using SPSS version 25.

**Results:**

One hundred drug users were recruited into the study. Overall, 7% of the drug users had HBV infection. 61% were found to be susceptible to HBV infection, 15% showed serological evidence of immunity from HBV vaccination and 1% developed natural immunity from HBV exposure. Significant risk factors for HBV infection were age less than 20 years, young age (*≤* 20 years) at drug initiation, being single, injecting drugs more than or equal to 20 times per month and injecting with used syringes.

**Conclusion:**

This study shows high intermediate endemicity of HBV infection among drug users, low vaccination uptake, and high susceptibility to HBV infection. This calls for the urgent inclusion of drug users in national HBV vaccination campaigns and the adoption of the World Health Organization’s recommendations on the prevention of viral hepatitis among people who inject drugs.

## Introduction

Hepatitis B virus infection is a global health challenge that jeopardizes public health and security. An estimated 296 million people worldwide are living with chronic HBV infection, and each year 1.5 million new infections and 1.2 million fatalities take place [1, 2]. Hepatitis B virus infection can result in chronic hepatitis, cirrhosis, hepatocellular cancer, and even mortality. The majority of HBV infections occur in Asia and Africa’s low- and middle-income nations. With an estimated overall prevalence of 12.2% in the general population, Nigeria, the most populous nation in sub-Saharan Africa, carries a heavy burden of this infectious disease [3]. Certain subgroups, such as drug users, suffer a higher risk of HBV infection than the overall population due to some risk factors related to their lifestyle and activities.

An estimated 284 million people globally used drugs at least once in the preceding 12 months in 2020 [4]. The problem of drug usage is widespread, and throughout Africa, it has been seen to be getting worse. As the continent continues to be a major transit hub for cocaine in the West, heroin in the East, and cannabis in the North, drug use in Africa is expected to increase by 40% by the year 2030 [4, 5]. The use of illicit drugs is illegal in Nigeria, a nation in West Africa, and offenders face 15–25 years in prison if found guilty [6]. Despite this, several studies have found that young people use drugs at a significant rate, with cannabis being the most popular drug [6, 7]. Blood-borne infectious illnesses are highly contagious and are most common in the first five years of drug usage [8]. This unique demographic maintains a quiet pool for HBV transmission in any situation and contributes 20% of the global burden of HBV infection.

Universal health coverage is emphasized as a crucial strategy in the elimination of viral hepatitis [1]. However, despite the HBV vaccine’s inclusion in the National Program on Immunization since 2004, no programs expressly target the prevention and treatment of HBV infection among high-risk populations, such as drug users [9]. Drug users still struggle to get access to comprehensive HBV treatment because of stigma, prejudice, a lack of understanding about the disease, access restrictions to medical care, and socioeconomic constraints. This in turn puts the World Health Organization’s goal of eradicating Hepatitis B Virus infection by the year 2030 in jeopardy.

There is a dearth of published information explicitly addressing the prevalence of HBV infection among drug users in Nigeria. The HBV immune status of drug users was not taken into account by the available studies, despite the fact that this information is crucial for guiding evidence-based treatments and adjusting preventative measures. This work aims to determine the prevalence, immune status, and related risk factors for HBV infection among illegal drug users in Enugu State, Nigeria.

## Materials and methods

### Study design, area, and population

This cross-sectional study was conducted from January to June 2018 in Enugu State. Enugu State is one of five states in the Southeastern zone of Nigeria. The state covers an area of 7,161 km^2^ with an estimated population of 3,267,837 (2006 census). Enugu State is one of the main transit routes into the Southeast and South-south zones of Nigeria. It harbours an international airport for the easy movement of people and goods both within and outside the country. Numerous academic institutions are located in the state, which draws a diverse group of young people from all over the nation. Drug users recruited into the study were members of drug bunks. Drug bunks are joints/hostel-like facilities where illicit drugs are supplied and sold. The drug bunks used for this study were purposively selected by YORDEL Africa, a non-governmental organization working with drug users in the state.

### Sample size determination

Using the method by Onwuasigwe et al. [10]. at a 95% confidence interval and an estimated prevalence of 7.8% for Hepatitis B virus infection among people who inject drugs in Lagos State, Nigeria, a representative sample size was established [11].

$${\rm{n = }}{{\rm{Z}}^{\rm{2}}}{\rm{pq/}}{{\rm{d}}^{\rm{2}}}{\rm{ = }}{{\rm{Z}}^{\rm{2}}}{\rm{p}}\left( {{\rm{1 - p}}} \right){\rm{/}}{{\rm{d}}^{\rm{2}}}$$

n = minimum sample size.

Z = normal standard deviate (1.96 for 95% confidence interval).

p = estimated prevalence of Hepatitis B infection among IDUs in Lagos State, Nigeria 7.8% [11].

q = proportion of the population that does not have the characteristic (that is 1-p).

d = precision or sampling error (5%).

$$\begin{array}{l}{\rm{n = }}\,{\rm{1}}{\rm{.96}}\,{\rm{ \times }}\,{\rm{1}}{\rm{.96}}\,{\rm{ \times }}\,{\rm{0}}{\rm{.078}}\,{\rm{ \times }}\,\left( {{\rm{1 -- 0}}{\rm{.078}}} \right)\,{\rm{/}}\,{\rm{0}}{\rm{.05}}\, \times \,{\rm{0}}{\rm{.05}}\\{\rm{ = }}\,{\rm{3}}{\rm{.8416}}\, \times \,{\rm{0}}{\rm{.078}}\, \times \,{\rm{0}}{\rm{.922}}\,{\rm{/}}\,{\rm{0}}{\rm{.0025}}\,{\rm{ = }}\,{\rm{110}}\end{array}$$

Finite correction for a known population of less than 10,000.

$${\rm{nf = }}\frac{{\rm{n}}}{{{\rm{1}}\,{\rm{ + }}\,{\rm{n/N}}}}$$

n = minimum sample size (110).

N = Total population from NGO records (400).

$${\rm{ = }}\frac{{110}}{{1 + 110/400}} = 86$$

Adjusting for 10% non-response rate.

$${\rm{NR = }}\frac{{\rm{n}}}{{{\rm{1 - NR}}}}$$

$${\rm{NR = }}\frac{{{\rm{86}}}}{{{\rm{1}} - {\rm{0}}{\rm{.1}}}} = 96$$

Following finite correction for a population less than 10,000 and adjusting for 10% non-response rate, the minimum sample size calculated for this study was 96. However, 100 drug users were recruited.

### Sampling technique

Drug users were enrolled in the study using the Privileged Access Interviewer (PAI) method [12], In this method people who inject drugs (PWID) or have previously used drugs interviewed the drug users. They identified and recruited the study population using the PAI approach with the assistance of a peer educator from the non-government organization (NGO) working with the study population. A standard sum was given to each participant in the study to cover the cost of transport.

### Inclusion criteria

1. Persons identified as drug users irrespective of age.
2. Respondents who give consent to participate in the study.

### Exclusion criteria

1. Critically ill persons.
2. Persons with severe mental conditions and unable to give consent.

### Data collection

Written and/or informed consent was sought from each participant prior to recruitment into the study. Simple English (primary school level) was used in the consent form and translated into the local Igbo language for participants with no formal education and those who preferred the local language. For participants under 18 years, the consent to participate in the study was obtained from their parent or legal guardian. All participants were then given pre-tested structured questionnaires to collect data on socio-demographics and risk factors for HBV infection.

### Sample collection

Five millilitres of blood was drawn from each participant aseptically and transferred into sterile plain test tubes. This was allowed to clot and then centrifuged at 3,000 g for 10 min, after which serum was transferred into clean cryovials and stored at -20°C.

### Laboratory analysis

Serum samples were processed after being allowed to reach room temperature. Using immunochromatographic HBV seromarker panels (Micropoint Bioscience Inc, California, USA), tests for HBV and seromarkers were conducted. The results were read within five minutes.

For HBsAg, HBsAb, and HBeAb, a negative result was indicated by only one purple bar in the control zone. A purple bar present in the test zone but absent in the control zone was regarded as an invalid result, while two purple bars, one in the control zone and the other in the test zone, signalled a positive result. For HBeAb and HBcAb, a positive result was indicated by only one purple bar in the control zone, a negative result by two purple bars, one in the control zone and the other in the test zone, and an invalid result by a purple bar present in the test zone but absent from the control zone.

### Statistical analysis

The Statistical Package for Social Sciences (SPSS) version 25 was used for all statistical analysis. Data analysis and variable comparisons were performed using descriptive statistical methods (mean, median, standard deviation), paired student t-tests to compare means, Spearman’s correlation, and multivariate logistic regression. *P* value < 0.05 was considered statistically significant.

### Post-test intervention

The test results were given to the participants. When HBV infection was discovered, individuals were counselled by the peer educator and directed to the closest tertiary medical centre for further management.

## Results

One hundred drug users were recruited into the study, of which 86 (86%) were males. The majority of participants, 44 (44%) were between the ages of 20 and 29, and 42 (42%) of the drug users had only secondary education as their highest level of schooling. 60% of respondents were self-employed and there was an equal distribution of single and married persons (1:1). (Table 1).

**Table 1:** Socio-demographic characteristics of study participants

Characteristic	Frequency (*n* = 100)	Percentage (%)
**Gender**		
Male	86	86
Female	14	14
**Age**		
10–19	5	5
20–29	44	44
30–39	34	34
40–49	11	11
*≥* 50 years	6	6
**Education**		
No formal education	28	28
Primary	11	11
Secondary	42	42
Tertiary/Postgraduate	19	19
**Employment status**		
Student	14	14
Self-employed/ Business owner	60	60
Public servant	4	4
Unemployed	21	21
Retired	1	1
**Marital status**		
Single	49	49
Married	50	50
Divorced/separated	1	1

Overall, 7% of the drug users had HBV infection. 61% of drug users were found to be susceptible to HBV, compared to 15% who showed evidence of immunity from HBV vaccination and 1% who developed natural immunity from HBV exposure. (Table 2)

**Table 2:** Serological markers of hepatitis B virus infection among drug users

HBsAg+	HBsAb+	HBeAg+	HbeAb+	HbcAb+	Frequency	Interpretation
7	0	3	4	7	7	HBV infection
0	0	0	0	0	61	Susceptible
0	1	0	1	1	1	Natural immunity
0	15	0	0	0	15	Vaccination
0	0	0	6	16	16	Past infection/ Possible occult infection
**7**	**16**	**3**	**11**	**24**	**100**	**Total**

**Fig. 1:**
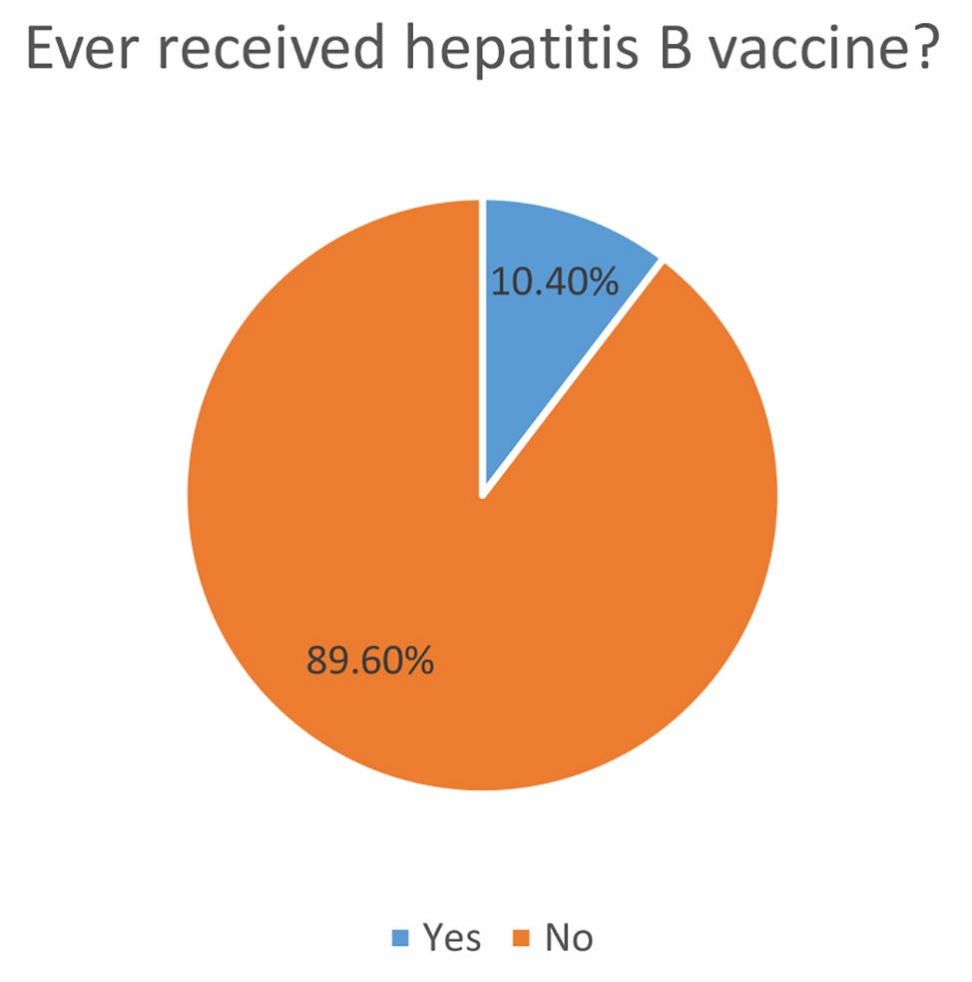
Pie chart showing the proportion of people who inject drugs who have received hepatitis B vaccine

Only 7(10.4%) of the 100 drug users who took part in the study said they had gotten the hepatitis B virus vaccination. (Fig. 1)

Using multivariate logistic regression, risk factors associated with increased chances of contracting hepatitis B virus infection include male gender (AOR = 1.214, *P* = 0.003), age group 10–19 years (AOR = 2.646, *P* = 0.015), single status (AOR = 3.859, *P* = 0.049), age at first drug use ≤ 20 years (AOR = 3.073, *P* = 0.002), frequency of drug injection/ month ≥ 20 times/ month (AOR = 2.087, *P* = 0.011), and drug injection with previously used syringes (AOR = 2.030, *P* < 0.05). The risk of HBV infection was lower in people without formal education. (Table 3).

**Table 3:** Multivariate analysis of factors associated with HBV infection

Variable	Number of HBV positive	AOR	*P* - value
Gender (male)	7/86	1.214	0.003
Age (10-19years)	2/5	2.646	0.015
Occupation (student)	2/14	0.221	0.124
Education (no formal education)	5/28	0.441	0.019
Marital status (single)	7/49	3.859	0.049
Sharing of cotton swabs, arm ties and injection water	1/13	0.977	0.592
Previous blood transfusion	2/16	0.015	0.787
Age at first drug injection (≤ 20 years)	6/45	3.073	0.002
Frequency of drug injection/ month (≥ 20 times/ month)	4/26	2.087	0.011
Drug injection with used syringes	3/16	2.030	0.026
Heterosexual intercourse	7/86	0.012	0.429
Anal sex	1/15	0.207	0.443

## Discussion

Hepatitis B virus infection is a significant problem globally and Nigeria is not exempted. Despite the availability of the HBV vaccine in the country, drug users, a high-risk group who continue to fuel the silent transmission of HBV infection in all settings, are often disregarded in HBV preventive programmes.

The HBV prevalence of 7% in the current study reflects high intermediate endemicity of HBV among drug users and this implies high rate of HBV transmission in the study population. This figure is lower than the HBV prevalence of 9.5% found in a comprehensive review and meta-analysis of HBV infection in the nation [13]. Given that the Southeastern region of Nigeria has the lowest reported prevalence of HBV among the six geopolitical zones in the country, this may be related to the geopolitical zone where the study was carried out. This could be further explained by increasing vaccination rates, better educational systems, and more use of learning opportunities in the region [13, 14]. 

The HBV prevalence in this study is comparable with the prevalence reported among male IDUs in Lagos State, where it was 7.8% [11]. The prevalence of HBV in West Africa was reported to be 7.8% in a report by the UN Office on Drugs and Crime [15]. In Mozambique, a higher HBV prevalence of 32.8% was recorded among drug users who inject substances (PWID) [16]. On the other hand, drug users in Iran, Turkey, and Kuwait had lower HBV prevalences of 4.8%, 2.6%, and 0.38%, respectively [17–19]. This may be due to efficient public health initiatives, the accessibility of diagnostic and treatment facilities, and the widespread use of vaccines in wealthy nations [17]. 

Despite the availability of the HBV vaccine in Nigeria and the WHO recommendation that drug users should be scheduled for quick immunization regimens, only 7 (10.4%) of drug users in the study reported having ever received the HBV vaccine. However, 15% of drug users in the study had serological proof of HBV vaccination while about two-thirds of individuals were still at risk for HBV infection. The disparity between reported vaccine uptake and HBV immune status among participants may be attributable to ignorance and low awareness of the HBV vaccine among drug users. In Australia and Germany, similar poor HBV vaccine uptake among drug users has been documented [20, 21]. Comparable results have been found in other high-risk populations [2, 22]. The failure to design tailored programs for HBV vaccination in particular populations, the cost of the vaccine, as well as the criminality and stigma associated with drug use, may all contribute to the under-vaccination of drug users [8, 23]. Drug users must be targeted if HBV epidemics are to be controlled. Thus, it has been demonstrated that offering incentives to enhance vaccine uptake and completion is economically viable for the utilization of limited public health resources, in addition to adopting WHO guidelines for quick immunization schedules [24]. 

Age less than 20 years and age at first drug use less than or equal to 20 years were highly predictive of HBV infection in drug users. Young people may underestimate their vulnerability to disease, be more prone to engage in risky sexual practices and show poor adherence to healthy lifestyle practices, all of which may contribute to this [8, 24]. In this study, additional risk factors for HBV infection were being single, injecting drugs more than or equal to 20 times per month and using syringes that had already been used.

Drug users’ risk of contracting HBV infection was observed to be higher among men and single status individuals. This could be the case because unmarried men were more likely than married men to have several sexual partners, participate in transactional sex and relocate in pursuit of better employment possibilities [25, 26]. 

The low percentage of female drug users in this study is consistent with findings from other research on drug users [16, 18, 27, 28]. This has been related to socioeconomic and cultural variables. In many African communities, traditional roles and cultural norms are respected; as a result, people who inject drug users may face greater shame than their male counterparts. As a result, there are “closet drug users” who might be overlooked in studies and preventive measures for drug users.

The study’s limitations include the small sample size since the community was difficult to contact and the inability to distinguish between those with acute and chronic infections because IgG HbcAb and IgM HbcAb were not measured. Additionally, immunochromatographic test panels were used and these have lower sensitivity and specificity when compared to the gold standard, DNA PCR.

## Conclusion

In our study, drug users have a high intermediate endemicity of HBV infection. Additionally, there is a low rate of HBV vaccination uptake and a high rate of susceptibility to HBV infection. Age less than 20 years, young age (*≤* 20 years) at drug initiation, being single, injecting drugs more than or equal to 20 times per month and injecting with used syringes were all determined to be significant risk factors for HBV infection.

It is urgent to include drug users in national HBV vaccination campaigns and adopt the World Health Organization’s recommendations on the prevention of viral hepatitis among people who inject drugs because of the high risk of HBV infection and low vaccination uptake among illicit drug users. To address the problem of drug usage, Nigeria must use a multisectoral and public health approach. This entails implementing targeted, evidence-based strategies like health education, screening, rapid HBV vaccination regimen, incentives to encourage the uptake of the HBV vaccine, and harm reduction strategies for drug users because these can significantly impact their health and the risk of spreading the disease to others [2, 24, 29]. 

## Data Availability

The corresponding author will provide the datasets used and/or analyzed during this investigation upon reasonable request.

## Abbreviations

HBV: Hepatitis B virus
HbcAb: Hepatitis B core antibody
HBeAg: Hepatitis B envelope antigen
HBeAb: Hepatitis B envelope antibody
HIV: Human immunodeficiency virus
IDU: Injecting drug users
NGO: Non-governmental organization
PAI: Privileged access interviewer
PWID: People who inject drugs
WHO: World health organization

## References

[1] World Health Organization. Global health sector strategies on, respectively, HIV, viral hepatitis and sexually transmitted infections for the period 2022–2030. Accessed 24 Jul 2023. file:///C:/Users/USER/Downloads/9789240053779-eng.pdf

[2] Dan-Nwafor CC, Adeoye I, Aderemi K, Onuoha M, Adedire E, Bashorun A et al. Serological markers and risk factors associated with hepatitis B virus infection among federal capital territory prison inmates, Nigeria: should we be concerned? Blackard JT, editor. PLOS ONE. 2021;16(3):e0248045. 10.1371/journal.pone.0248045 PMC7951833 33705419

[3] Olayinka AT, Nasidi A, Gidado S, Sha’aibu S, Balogun MS, Oyemakinde A (2016). Seroprevalence of Hepatitis B infection in Nigeria: A National Survey. Am J Trop Med Hyg.

[4] United Nations Office on Drug and Crime. World Drug Report. 2022. Accessed 9 Mar 2023. Available from file:///C:/Users/OKONKWO IBUCHUKWU/Downloads/WDR22_Booklet_1.pdf

[5] United Nations Office on Drug and Crime. World Drug Report. 2021. Accessed 9 Mar 2023. https://www.unodc.org/res/wdr2021/field/WDR21_Booklet_1.pdf

[6] National Drug Law Enforcement Agency (Disposal of Forfeited Asset and Properties) Regulations. Accessed 9 Mar 2023. https://nigeriatradeportal.fmiti.gov.ng/media/NDLEA Act.pdf

[7] Jatau AI, Sha’aban A, Gulma KA, Shitu Z, Khalid GM, Isa A (2021). The Burden of drug abuse in Nigeria: a scoping review of Epidemiological studies and Drug laws. Public Health Rev.

[8] Seal KH, Ochoa KC, Hahn JA, Tulsky JP, Edlin BR, Moss AR (2000). Risk of hepatitis B infection among young injection drug users in San Francisco: opportunities for intervention. West J Med.

[9] Ikobah J, Okpara H, Elemi I, Ogarepe Y, Udoh E, Ekanem E (2016). The prevalence of hepatitis B virus infection in Nigerian children prior to vaccine introduction into the National Programme on immunization schedule. Pan Afr Med J.

[10] Onwuasigwe C (2004). Determination of sample size. Medical Research Project: a practical guide.

[11] Tun W, Vu L, Adebajo SB, Abiodun L, Sheehy M, Karlyn A (2013). Population-based prevalence of hepatitis B and C virus, HIV, syphilis, gonorrhoea and chlamydia in male injection drug users in Lagos, Nigeria. Int J STD AIDS.

[12] Griffiths P, Gossop M, Powis B, Strang J (1993). Reaching hidden populations of drug users by privileged access interviewers: methodological and practical issues. Addiction.

[13] Ajuwon BI, Yujuico I, Roper K, Richardson A, Sheel M, Lidbury BA (2021). Hepatitis B virus infection in Nigeria: a systematic review and meta-analysis of data published between 2010 and 2019. BMC Infect Dis.

[14] Aghedo I, Eke S (2013). From alms to arms: the Almajiri Phenomenon and Internal Security in Northern Nigeria. Korean J Policy Stud.

[15] United Nations Office on Drugs and Crime. World drug report 2022_Annex. Accessed 9 Mar 2023. https://www.unodc.org/unodc/en/data-and-analysis/wdr2022_annex.html

[16] Semá Baltazar C, Boothe M, Kellogg T, Ricardo P, Sathane I, Fazito E (2020). Prevalence and risk factors associated with HIV/hepatitis B and HIV/hepatitis C co-infections among people who inject drugs in Mozambique. BMC Public Health.

[17] Rostam-Abadi Y, Rafiemanesh H, Gholami J, Shadloo B, Amin-Esmaeili M, Rahimi-Movaghar A (2020). Hepatitis B virus infection among people who use drugs in Iran: a systematic review, meta-analysis, and trend analysis. Harm Reduct J.

[18] Karabulut N, Bulut Y, Telo S (2015). Frequency of Hepatitis B and C viruses, and HIV among Drug addicts in the Eastern Anatolia, Turkey. Jundishapur J Microbiol.

[19] Altawalah H, Essa S, Ezzikouri S, Al-Nakib W (2019). Hepatitis B virus, hepatitis C virus and human immunodeficiency virus infections among people who inject drugs in Kuwait: a cross-sectional study. Sci Rep.

[20] White B, Dore GJ, Lloyd A, Rawlinson W, Maher L (2012). Ongoing susceptibility to hepatitis B virus infection among people who inject drugs in Sydney. Aust N Z J Public Health.

[21] Haussig JM, Nielsen S, Gassowski M, Bremer V, Marcus U, Wenz B (2018). A large proportion of people who inject drugs are susceptible to hepatitis B: results from a bio-behavioural study in eight German cities. Int J Infect Dis.

[22] Aniche OMC, Orabueze IN, Nwafia IN, Ihezuo JU, Chinaka CB, Egbe KA (2022). Prevalence of Hepatitis B Virus Seromarkers in Female Sex workers in Enugu State, Nigeria. Venereology.

[23] Ranjan A, Shannon K, Chettiar J, Braschel M, Ti L, Goldenberg S (2019). Barriers and facilitators to hepatitis B vaccination among sex workers in Vancouver, Canada: implications for integrated HIV, STI, and viral hepatitis services. Int J Infect Dis.

[24] Hwang LY, Grimes CZ, Tran TQ, Clark A, Xia R, Lai D (2010). Accelerated hepatitis B vaccination schedule among drug users: a randomized controlled trial. J Infect Dis.

[25] Zembe YZ, Townsend L, Thorson A, Ekström AM (2013). Money talks, bullshit walks interrogating notions of consumption and survival sex among young women engaging in transactional sex in post-apartheid South Africa: a qualitative enquiry. Globalization Health.

[26] Shisana O, Risher K, Celentano DD, Zungu N, Rehle T, Ngcaweni B, Evans MG (2016). Does marital status matter in an HIV hyperendemic country? Findings from the 2012 South African National HIV Prevalence, incidence and Behaviour Survey. AIDS Care.

[27] Degenhardt L, Peacock A, Colledge S, Leung J, Grebely J, Vickerman P (2017). Global prevalence of injecting drug use and sociodemographic characteristics and prevalence of HIV, HBV, and HCV in people who inject drugs: a multistage systematic review. Lancet Glob Health.

[28] James BO, Olotu SO, Ayilara OO, Arigbede OO, Anozie GI, Ogiku HO (2019). Drug treatment presentations at a treatment centre in southern Nigeria (2015–2018): findings and implications for policy and practice. Niger Postgrd Med J.

[29] World Health Organization. Guidance on prevention of viral hepatitis B and C among people who inject drugs 2012. Accessed 13 Jul 2023. https://apps.who.int/iris/bitstream/handle/10665/75357/9789241504041_eng.pdf;jsessionid=81A164A64DC05CF7361A56F9E0950AAA?sequence=1 23805439

